# Mechanisms of Non-Alcoholic Fatty Liver Disease in the Metabolic Syndrome. A Narrative Review

**DOI:** 10.3390/antiox10020270

**Published:** 2021-02-10

**Authors:** Luca Rinaldi, Pia Clara Pafundi, Raffaele Galiero, Alfredo Caturano, Maria Vittoria Morone, Chiara Silvestri, Mauro Giordano, Teresa Salvatore, Ferdinando Carlo Sasso

**Affiliations:** 1 Department of Advanced Medical and Surgical Sciences, University of Campania Luigi Vanvitelli, Piazza Luigi Miraglia 2, 80138 Naples, Italy; luca.rinaldi@unicampania.it (L.R.); piaclara.pafundi@unicampania.it (P.C.P.); raffaele.galiero@unicampania.it (R.G.); alfredo.caturano@unicampania.it (A.C.); lifetime89@hotmail.it (C.S.); mauro.giordano@unicampania.it (M.G.); 2 Department of Experimental Medicine, Section of Microbiology, University of Campania Luigi Vanvitelli, Piazza Luigi Miraglia 2, 80138 Naples, Italy; mariavittoria.morone@unicampania.it; 3 Department of Precision Medicine, University of Campania Luigi Vanvitelli, Via De Crecchio 7, 80138 Naples, Italy; teresa.salvatore@unicampania.it

**Keywords:** NAFLD, MAFLD, metabolic syndrome, insulin resistance, epigenetics, oxidative stress

## Abstract

Non-alcoholic fatty liver disease (NAFLD) and metabolic syndrome (MS) are two different entities sharing common clinical and physio-pathological features, with insulin resistance (IR) as the most relevant. Large evidence leads to consider it as a risk factor for cardiovascular disease, regardless of age, sex, smoking habit, cholesterolemia, and other elements of MS. Therapeutic strategies remain still unclear, but lifestyle modifications (diet, physical exercise, and weight loss) determine an improvement in IR, MS, and both clinical and histologic liver picture. NAFLD and IR are bidirectionally correlated and, consequently, the development of pre-diabetes and diabetes is the most direct consequence at the extrahepatic level. In turn, type 2 diabetes is a well-known risk factor for multiorgan damage, including an involvement of cardiovascular system, kidney and peripheral nervous system. The increased MS incidence worldwide, above all due to changes in diet and lifestyle, is associated with an equally significant increase in NAFLD, with a subsequent rise in both morbidity and mortality due to both metabolic, hepatic and cardiovascular diseases. Therefore, the slowdown in the increase of the “bad company” constituted by MS and NAFLD, with all the consequent direct and indirect costs, represents one of the main challenges for the National Health Systems.

## 1. Introduction

Non-alcoholic fatty liver disease (NAFLD) and metabolic syndrome (MS) are two different entities sharing common clinical and physio-pathological features. MS is defined as the coexistence of several factors predisposing to cardiovascular (CV) disease and it is diagnosed at the presence of at least three conditions among the following: Abdominal obesity (>102 cm in men and >88 in women), increased triglycerides levels (>1.69 mmol/L), reduced HDL cholesterol levels (<1.03 mmol/L in men, <1.29 mmol/L in women), increased blood pressure (>130/85 mmHg), and hyperglycemia (>5.56 mmol/L or ongoing anti-hyperglycemic therapy) [1]. NALFD includes several histological pictures, ranging from the mere liver steatosis till overt liver cirrhosis [2]. Both MS and NAFLD are metabolic disorders with a high clinical relevance and important health and social implications.

MS affects almost the 20–30% of general population and half of the elderly, with differences between ethnic groups and economic and geographical areas. Hispanic and Indians seem particularly susceptible of this condition [3]. NAFLD, indeed, has been frequently related to obesity, mostly abdominal, diabetes, and dyslipidemia, thus being considered the hepatic manifestation of MS [4].

Thanks to the large frequency of blood tests and ultrasound examinations among asymptomatic individuals, NAFLD and MS have currently reached a prevalence of epidemic proportions, turning out as the most common causes of chronic liver disease in Western countries [5,6,7], with an estimated prevalence around the 20–30% in the adult population and peaks of the 70–90% among obese and diabetics.

Beyond epidemiologic features, MS and NAFLD share several pathogenetic features, with insulin resistance (IR) as the most relevant [8]. IR plays a crucial role in MS and it is tightly associated with an increase of visceral adipose tissue mass. This latter shares a directly proportional release of mediators from adipocytes [9], which inhibit the sensitivity to insulin action such as leptin and resistin [10]. Conversely, adiponectin seems to exert a role in facing systemic IR [11], and the subsequent development of NAFLD [12]. IR, indeed, determines an accumulation of free fatty acids (FFAs) inside the liver, due to an increased hepatic lipogenesis and the missed suppression of lipolysis of the adipose tissue [13]. The accumulation of intrahepatic fat, in turn, determines a modification of insulin signaling pathways, thus worsening the systemic state of IR [14]. Such a behavior renders these two conditions closely interconnected.

Along with IR, also genetic and epigenetic factors, diet, lifestyle, mitochondrial dysfunction, low degree chronic inflammation, adipose tissue dysfunction, oxidative stress and of endoplasmic reticulum (ER), microbiota and constitutive immunity may be involved [15]. These elements, identified under a complex theory named the “multiple parallel hits hypothesis” [16], act in a convergent and synergic way in the hepatocyte, thus inducing the damage. NAFLD, particularly steatohepatitis, is associated with an increased mortality risk. Large evidence leads to consider it as a risk factor for CV disease, regardless of age, sex, smoking habit, cholesterolemia and the remaining elements of MS [17]. Therapeutic strategies remain still unclear, but lifestyle modifications (diet, physical exercise, and weight loss) determine an improvement in IR, the eventually present MS and of both clinical and histologic liver picture.

## 2. Methods

We ran an electronic search in PubMed/MEDLINE, Scopus, and Web of Science for literature updated to 20 January 2021. A combination of the following keywords was used: (1) “liver steatosis” OR “NASH” AND “Insulin Resistance” OR “Metabolic Syndrome” OR “Diet” OR “Microbiota” OR “Genetics” or (2) “NAFLD” OR “MAFLD” AND “Insulin Resistance” OR “Metabolic Syndrome” OR “Diet” OR “Microbiota” OR “Genetics”.

The full-text articles of all potential studies were evaluated. Moreover, we conducted a manual search of references to relevant articles, including older human studies and animal studies, to find additional publications that might have missed through electronic searches only. Articles for which the full text was not accessible or not available in English, French and Spanish excluded. Duplicate articles were removed, and a first screening was performed by reading only the titles and abstracts of the studies.

## 3. NAFLD and MAFLD

From an anatomopathological point of view, liver steatosis is defined either as an accumulation of lipids in the liver higher than 5–10% of the organ weight or by the presence of lipid droplets in more than 5% of hepatocytes [18]. NAFLD is a clinical condition characterized by an excessive liver fat accumulation, associated with IR, and it is more in depth defined by the presence of steatosis in >5% of hepatocytes according to histological analysis or by Proton density fat fraction (PDFF) calculated by magnetic resonance imaging (MRI) [19]. NAFLD, indeed, is a condition of steatosis in the absence of clinical/anamnestic evidence of ethyl alcohol abuse or other known causes of liver damage. NAFLD is responsible for various pictures ranging from benign (simple hepatic steatosis), to non-alcoholic steatohepatitis (NASH), in which the intra-hepatocytic accumulation of triglycerides is further accompanied by an intense necro-inflammatory activity [20], till overt liver cirrhosis and all its allied complications (e.g., liver failure, portal hypertension, and hepatocellular carcinoma (HCC)) [2,21].

Currently, NAFLD has become the most frequent cause of chronic liver disease, to the detriment of the forms of infectious etiology which, on the contrary, show a progressively decreasing trend. As well, NAFLD is among the main causes of cirrhosis and HCC in Western countries [22], with a growing prevalence tightly linked to modifications of lifestyle habits, emblematic of Western culture, with a propensity towards overfeeding and physical inactivity.

NAFLD is also often associated with obesity, especially abdominal, diabetes and dyslipidemia and it is strictly linked to the increase of several clinical and biological markers of IR. Actually, NALFD is mainly present in patients with MS, where it represents the hepatic organ manifestation.

Recently, several international hepatologists have proposed to rename NAFLD as MAFLD (metabolic associated fatty liver disease) [23]. The new term should replicate the tight association between fatty liver and overfeeding, physical inactivity and metabolic conditions (e.g., type 2 diabetes (T2DM), hypertension, dyslipidemia, and obesity) [24,25]. The new diagnostic criteria proposed for MAFLD are based on evidence of fatty liver and coexistence of overweight/obesity (BMI > 25 kg/m^2^ in white subjects or >23 kg/m^2^ in the Asian population) or T2DM [22]. Indeed, in subjects with a normal weight a crucial role is played by the co-presence of liver steatosis and two risk factors related to metabolic dysregulation: Waist circumference ≥ 108 cm in White men and ≥88 cm in women (≥90 cm and ≥80 cm, respectively, among Asians); prediabetes (fasting glycaemia 5.56–6.94 mmol/L or glycaemia levels between 7.78–11.06 mmol/L after oral glucose tolerance test (OGTT) or HbA1c between 39–46%); inflammation (serum C-reactive protein levels > 2 mg/L), blood pressure ≥ 130/85 mmHg; decrease in HDL cholesterol levels < 1.03 mmol/L in men and <1.29 mmol/L in women; increase in plasma triglyceride levels ≥ 1.69 mmol/L or specific drug treatment, and HOMA index ≥ 2.5 [23].

An interesting aspect of MAFLD definition is that intake of alcohol and other chronic liver diseases are not essential for the diagnosis. In fact, no alcohol intake threshold has been defined yet and, as well, no consideration has been focused on the interindividual variability in reply to alcohol consumption based on age, sex, ethnicity, duration of alcohol consumption, timing (ongoing vs remote) and genetic susceptibility. This renders the definition of an absolute alcohol intake threshold for a given individual unreliable. Moreover, often the patient tends to underestimate the daily alcohol intake. Beyond this, recent data suggest the production of alcohol by some intestinal bacteria, thus contributing to liver damage [26]. Furthermore, large evidence suggests the presence of shared genetic factors in alcohol-associated fatty liver disease and metabolic dysfunction, with a fundamental role of the accumulation of liver lipids, which can be followed by liver inflammation and disease progression in both conditions [27,28,29,30,31].

**Figure 1:**
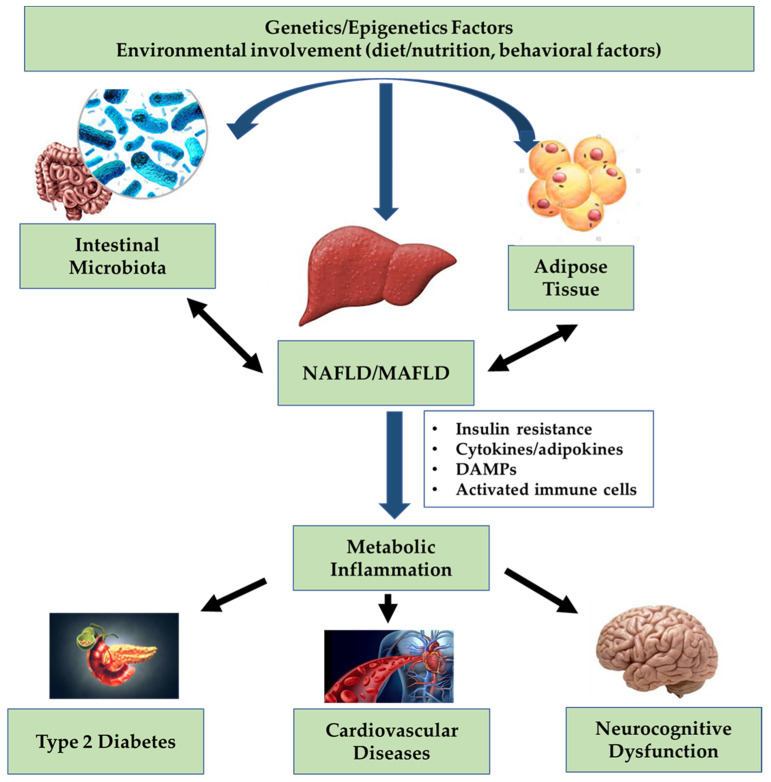
Interplay between liver and body targets in non-alcoholic fatty liver disease (NAFLD)/metabolic associated fatty liver disease (MAFLD).

Therefore, given the progresses in the knowledge of the role of metabolic dysfunction in triggering and promoting both development and progression of hepatic steatosis, it seems appropriate to introduce the new term MAFLD, with a series of “positive” criteria established to define the pathological condition (Figure 1). These criteria have been applied to real life and have made possible to better identify fatty liver disease at higher risk of progression [32,33,34].

## 4. Metabolic Syndrome and Insulin Resistance

The cornerstone of the MS is IR, which is closely related to visceral fat. Insulin resistance state occurring when cells in muscles, fat and liver do not respond well to insulin action. Subsequently, individuals display a difficult blood glucose metabolism as compared to the general population and the pancreas thus produces much more insulin to help glucose enter your cells.

Insulin is a pancreatic hormone secreted by the beta cells of the islets of Langerhans [35]. Once secreted, it can bind to the extracellular domain of its specific receptor, with the subsequent production of a conformational change and induction of phosphorylation of specific tyrosine residues on the intracytoplasmic domain of the insulin receptor (IRS) itself [36]. Hence, the receptor results activated and clips IRS substrates, in turn activated by tyrosine-phosphorylation processes. IRS molecules are thus able to activate the phosphatidylinositol 3-kinase (PI3K), which determines the translocation of glucose transporter protein type-4 (GLUT-4) from the cytoplasm to the cytoplasmic membrane, allowing the internalization of glucose bound to the receptor inside the cell. Activated PI3K can also exert anti-lipolytic effects, as well as activate fatty acid and glycogen synthases, thus brokering the anabolic effects typical of insulin. The phosphorylated IRS may also induce the activation of the RAS/MAP pathway, which is embroiled both in cell survival and stimulation of mitosis [37]. This behavior suggests that the regulation of these two biochemical cascades activated by insulin, i.e., regulation of intermediate metabolism and stimulation of cell growth and proliferation, may be untied. In fact, IR development during MS displays typical characteristics, such as impact on the processes of glucose cellular absorption, abolition of adipose tissue lipolysis and compromise of vasodilation, while there is no impairment of its action on growth and cytogenesis [38]. The selective inhibition of insulin action on metabolism and vasodilation could stand in an interference either at IRS or PI3K level [39]. Despite alterations of insulin/IRS bond, autophosphorylation and kinase activity processes, either IRS or PI3K/MAP kinase due to genetic changes [40,41], IR during MS mainly develops due to environmental factors related to a wrong lifestyle, typically described by an unbalance between caloric intake and energy consumption. This positive energy balance is at the basis of visceral obesity development. In fact, the relationship between insulin resistance and amount of visceral adipose tissue mass is directly proportional [9]. Moreover, weight loss has been reported to improve insulin sensitivity thanks to the reduction of visceral adipose tissue mass [42]. Hence, adipose tissue regulates insulin sensitivity in target tissues [43].

In fact, adipocytes have the role either of storing fatty acids in the form of triglycerides or releasing them again as FFASs and glycerol into the blood in relation to the body needs. However, circulating fatty acids, if highly present, can desensitize the target tissues to the action of insulin. When excess, FFAs induce the activation of a serine kinase protein capable to phosphorylate the serine residues on IRS and PI3K. This condition inhibits the phosphorylation of IRS and PI3K on their tyrosine residues and, consequently, their activation, thus determining a block in the transmission of the insulin signal and consequential IR [44].

Adipocytes also act secreting a variety of hormones, commonly known as adipokines. Among these, adiponectin stimulates the action of insulin in peripheral tissues [45], while leptin and resistin inhibit the sensitivity to insulin action [10]. Visceral obesity is linked to increased leptin levels and decreased adiponectin. Adipocytes also secrete molecules named chemokines, which can easily recruit macrophages within the adipose tissue. This is in turn guilty of an increase in tumor necrosis factor-alpha (TNF-α) levels [46]. TNF-α, such as resistin and other pro-inflammatory cytokines such (e.g., interleukin-6, IL-6), acts by enhancing IR occurrence and it is closely related to the degree of endothelial dysfunction [47]. Therefore, a wrong lifestyle may produce an excessively positive caloric balance, thus causing IR by enhancing visceral adipose tissue and consequently releasing a much higher level of FFAs, TNF-α and adipokines.

IR seems to exert a central role also in the pathogenesis of NAFLD. In fact, it determines an increase in hepatic lipogenesis and a lack of suppression of the lipolysis in the adipose tissue, thus producing an increase in the fatty acids flow inside the liver [13,48,49,50].

Patients with NAFLD also show increased de novo hepatic lipogenesis as compared to healthy controls, neither suppressed in fasting nor with higher FFA plasma levels [51]. Hepatic lipogenesis, active during IR, may be further induced by the activation of transcription factors such as sterol regulatory element-binding protein-1 (SREBP-1), carbohydrate-responsive element-binding protein (ChREBP) and peroxisome proliferator-activated receptor gamma (PPAR-γ) [52].

SREBP-1 is a transcription factor existing in different isoforms: SREBP-1c regulates de novo lipogenesis and is stimulated by insulin, while SREBP-2 is engaged in cellular cholesterol homeostasis [53]. ChREBP is instead activated by glucose and induces lipogenesis, though also providing more substrates for both triglycerides and FFAs synthesis.

**Figure 2:**
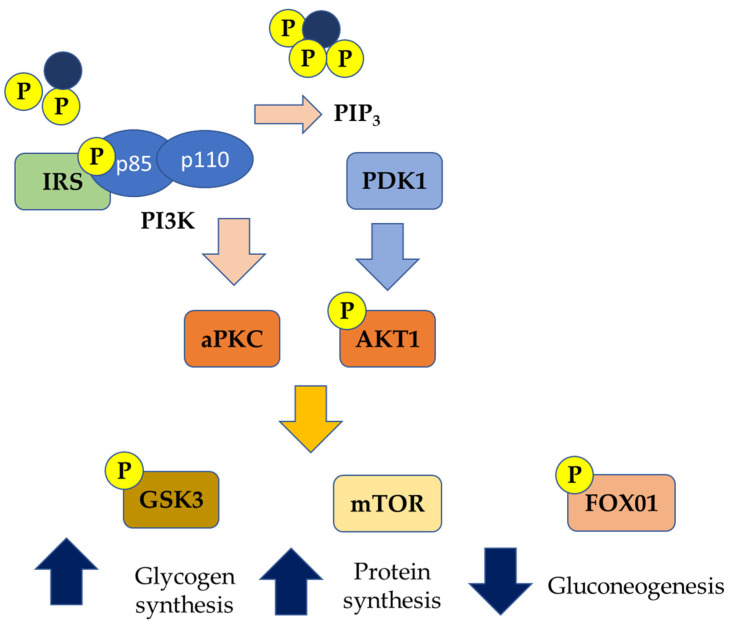
Genetic pathways of insulin resistance (PIP3: Phosphatidylinositol (3,4,5)-trisphosphate; aPKC: Activated protein kinase C; AKT1: Protein kinase B; GSK3: Glycogen synthase kinase 3; mTOR: mammalian target of rapamycin; FOXO1: Forkhead box protein O1; PDK1: 3-Phosphoinositide-dependent protein kinase 1; P: phosphorylated).

Among insulin receptors, IRS-2, once activated, can play as a regulator of SREBP-1c, affecting de novo lipogenesis. In IR conditions, IRS-2 is downregulated; hence SREBP-1c is overexpressed, thus stimulating lipogenesis [54]. In parallel, under IR conditions, FFAs beta-oxidation is inhibited, further favoring the hepatic accumulation of lipids [55] (Figure 2). Once accumulated in the liver, FFAs are able to induce alterations in the insulin signaling pathways by activating the serine kinase, subsequently contributing to the worsening of the systemic state of IR [14].

## 5. The Microbiota

Human body has been estimated to include about 10–100 trillion microbes, mostly located in the large intestine, for a maximum weight of 1.5 kg and more than 1000 bacterial types [56,57]. Despite the large microbial diversity, only 4 bacterial phyla govern intestine: Firmicutes, Bacteroidetes, Actinobacteria, and Proteobacteria, with Firmicutes and Bacteroidetes representing the 90% of all intestinal microbes. The sequencing of the 16S ribosomal RNA gene has allowed a clearer view of the diversity of the intestinal microbiota [58].

The normal intestinal microbiota can produce several substances beneficial for the health of the host regulating immunity, as well as by the integration of nutrition and homeostasis [59,60]. The gut microbiota interacts with the liver via the so-called “liver-gut” pathway, which involves some specific metabolites, such as bile acids (BA), lipopolysaccharides (LPS), and short-chain fatty acids (SCFA) [61]. Lipids and carbohydrates metabolism involve different types of bacteria and it is associated with the obesity related energy metabolism. Due to this reason, the intestinal microbiota and its metabolites can regulate most disorders related to energy metabolism, such as hyperlipidemia, atherosclerosis, diabetes, and inflammation) [62,63].

Changes in both internal and external host’s environment (e.g., diet, alcohol intake, antibiotics, and genetic factors) may alter the steadiness of the intestinal microbiota, which results in subsequent dysbiosis [64]. Currently, dysbiosis has been proven to significantly affect the pathogenesis of human liver diseases, particularly NAFLD and associated metabolic disorders [65]. The intestinal microbiota has been also reported to induce modifications of the expression of hepatic and intestinal genes tangled in the inflammatory, hormonal, and metabolic state in mice, while improving IR, a key characteristic of NAFLD [66]. Moreover, in a study on humans conducted by Vrieze and colleagues [67], infusion of allogenic gut microbiota has been demonstrated to improve sensitivity to peripheral insulin, whilst the introduction of a low-calories dietary regimen in obese individuals decreased the relative abundance of Firmicutes, while increasing that of Bacteroidetes [68]. More recently, emerging evidence has established that dysfunction of the intestine-liver axis, including alteration of mucosal permeability, bacterial proliferation, and intestinal dysbiosis, exert a significant impact on both development and progression of NAFLD [69]. Therefore, concentration of systemic endotoxin, increase in the intestinal epithelium permeability and levels of endogenous ethanol may result in an abnormal function of the intestine-liver axis in liver diseases. All these factors may also trigger the production of a cascade of cytokines, which activate the uncontrolled immune response at the basis of the release of multiple inflammatory mediators [70,71].

At the level of gut microbiota we can also observe the production of enzymes which catalyze the conversion of dietary choline into toxic compounds (e.g., methylamines). These can further attain the liver, where they are transformed into trimethylamine-N-oxide, which in turn can induce inflammation and liver damage [72]. Microbiota dysbiosis can thus endorse NASH through the reduction of choline levels and an increase in methylamine [73]. Moreover, other mechanisms seem implicated in promoting NAFLD development the intestinal microbiota. Particularly this latter appears able to alter bile acids metabolism, affecting the processes of de novo lipogenesis and VLDL export [74].

## 6. Oxidative Stress and Chronic Inflammation

NAFLD is a complex and multifactorial disease which involves several genetic, epigenetic and environmental factors. However, its pathogenesis has not been fully understood yet [75]. Due to the sensitivity to fat accumulation, the liver induces inflammation and cell death via a second pathogenic insult, which consequently translate into oxidative stress to finally result in NASH and fibrosis [76,77].

Currently, the more recent theory named the “multiple hit” hypothesis, involving several factors able to act in parallel, allows for a more fitting explanation of the pathogenesis. The most considerable factor contributing to the so-called “multiple strokes” is the oxidative stress, considered as the most involved in liver damage onset and disease progression in NAFLD [78,79]. Reactive oxygen species (ROS), including superoxidation radicals (O2^•−^) and hydrogen peroxide (H_2_O_2_), are endlessly formed inside the cells as by-products of energy metabolism in different liver cell-types [80]. Hepatic lipid overload induces the overproduction of oxidants by affecting several mechanisms that generate ROS. At high concentrations, ROS induce oxidative changes to cellular macromolecules (DNA, lipids, proteins, etc.) and increase the accretion of damaged macromolecules, which in turn determines liver damage [76,77]. Moreover, depending on ROS sources, cell types, and tissue environment, ROS signaling may play a crucial role in common physiological processes (e.g., regulation of cellular homeostasis, participation to a maladaptive response promoting metabolic dysfunction and inflammatory response) [81,82]. Therefore, there may exist a relationship between the mechanisms by which ROS promote the progression of NAFLD and both an arbitrary oxidative biomolecular damage and a dysregulation of redox signaling [83], although explicit molecular pathways have not been noticeably understood yet.

Mitochondrial dysfunction not only simplifies ROS production, though it also contributes to NAFLD progression by inducing hepatic inflammatory cytokines. Obesity, IR and adipokine/cytokine networks has been supposed to induce both the accumulation of hepatic fat and the development of NASH [84]. ROS, together with lipid peroxidation products, lead to an increase in the release of various cytokines (TNF-α, Fas ligand) which exert a crucial role in apoptosis, inflammation and fibrosis [76,85]. Besides the membrane damage caused by lipid peroxidation, we may also observe protein damage development as a result of oxidative stress. The aldehyde end-products of lipid peroxidation are well-known proinflammatory mediators, which may activate stellate cells, and thus produce an increase in collagen synthesis and, finally, development of liver fibrosis [86].

As a consequence of ROS-mediated mechanisms we may observe lipid peroxidation, the release of inflammatory cytokines and apoptosis. Biologically active lipid peroxidation products and cytokines work jointly to induce liver inflammation, and lead to the subsequent onset of various liver lesions related to NASH [76]. As a result of the upregulation of pro-inflammatory cytokines, then we observed the induction of an inflammatory response (e.g., TNF-α, interleukin-1 and IL-6). Inflammation molecules can thus exert a crucial role on the direction of both polymorphonuclear and mono-nuclear leukocytes into the inflamed tissues [87,88]. Moreover, an association of Kupffer cell activation and Fas-ligand death receptor expression with increased necrosis via caspase activation and Fas-ligand-mediated apoptosis has been observed [89,90].

TNF-α role on NASH is strengthened by an abnormal cytokine profile and an increased expression of its receptor in the liver [91]. Such conditions support the additional lipid peroxidation of mitochondrial membranes, thus producing a worsening of their function and subsequent induction of oxidative stress [87]. Adipose tissue exhibits a large deregulation of inflammation-related genes in patients affected by NASH of NADPH oxidase induction by TNF-α may also bring to inflammation through the expression of TNF receptor-1 and the activation of nuclear factor kappa B (NF-κB) [89,92]. In such a process, two main inflammatory pathways, JNK-AP-1 and IKK-NF-κB, exert a crucial role in the development of chronic inflammation occurring during NAFLD [93]. Jun *N*-terminal kinase (JNK) is a member of the mitogen-activated protein kinases, which are linked to the induction of apoptosis and the development of NASH.

NF-kB is both a transcription factor and a regulator of inflammation, and the subunit of its receptor IKK2 represents the main component which is fundamental for its activation during the acute inflammatory response [94]. Individuals with NASH display an endlessly activated NF-kB pathway [95]. The overexpression of IKK2 and the persistent activation of NF-kB in the hepatocytes produce a state of chronic inflammation and exacerbation of IR [96].

Hepatic exposure to increased levels of pro-inflammatory cytokines determines histological changes typical of NASH (e.g., hepatocyte necrosis and apoptosis, neutrophil chemotaxis, hepatic stellate cells (HSC) activation, and Mallory bodies production) [97]. In fact, obese patients show higher TNF-α and IL-6 serum levels which, on the other hand, may reduce, following weight loss [98]. However, onset of NASH may result in higher serum and hepatic levels of TNF-α, which seems associated with the histological gravity of liver damage [99].

## 7. Diet and Lifestyle

Diet, physical exercise and weight loss represent the only effective therapeutic aid for NAFLD [100,101], as recommended by both European Association for the Study of Liver (EASL) and American Association for the Study of Liver Disease (AASLD) guidelines [19,102]. Studies on NAFLD/NASH patients have demonstrated that a 7–10% reduction in body weight and regular aerobic physical activity lead to an improvement in both inflammation of liver tissues and fibrosis.

A healthy diet refers to the right balance between quantity and quality of food [103,104]. Beyond limiting the caloric intake, a correct balance between micro- and macro- nutrients is mandatory. As well, the reduction of the amount of saturated fats and carbohydrates such as fructose is equally important as the intake of lean proteins, fibers and polyunsaturated fatty acids (PUFA) [105,106].

### 7.1. Mediterranean Diet

The Mediterranean diet (MD) represents a nutritional model firstly originated in the countries surrounding the Mediterranean Sea. MD has thus been traditionally exerted by people settled in these regions. Although the MD model is extremely variable among countries and regions due to culture, ethnicity, religious and agricultural habits, it commonly includes nutrition with mainly unrefined grains, vegetables and fresh fruits, olive oil and nuts, fish, white meats and legumes in moderation, while red meat, processed meat and sweets were limited and red wine consumption had not be excessive [107].

Therefore, MD main features are represented by the beneficial profile of fatty acids, i.e., a low consumption of saturated fat and cholesterol and, on the contrary, a high feeding with mono-unsaturated fatty acid (MUFA), with a balanced PUFA omega-6/omega-3 ratio, as well as complex carbohydrates and fibers [108].

Many studies suggest that the anti-inflammatory and antioxidant properties of MD components may depict the basis of its protective effects. Particularly, the nutraceutical effect of bioactive and phytochemical compounds with antioxidant and anti-inflammatory capabilities (e.g., fibers, monounsaturated and omega-3 fatty acids and phytosterols) seem to exert a crucial role in the risk reduction of both NAFLD development and progression [109].

### 7.2. MD Mechanism: Antioxidant and Lipid Lowering Effects

The components producing MD positive effects are represented by polyphenols, vitamins and other biomolecules with anti-inflammatory properties and antioxidant effects. Whole grains, vegetables and fresh fruit, olive oil, nuts and red wine represent the main sources of polyphenols. These are a heterogeneous group of bioactive compounds, and comprise several water-soluble antioxidants, divided into flavonoids and non-flavonoids [110]. Flavonoids are ubiquitous polyphenolic compounds commonly known as main supplier of flavors and colors to fruits and vegetables, even though, due to their antioxidant and anti-inflammatory action, they can also exert hepatoprotective effects. Among non-flavonoids, resveratrol, found for example in red wine, stands out and exerts a hepatoprotective activity affecting the three interacting components of homeostasis (blood vessels, blood platelets and coagulation and the fibrinolytic system of plasma).

Vitamins and other substances (e.g., sylimarin 400 mg/day, Vitamin E 12 mg/day, *N*-acetyl cysteine 600 mg/day betaine 600 mg/day and selenium 81 µg/day), important MD components, may also be considered food antioxidants [111]. They reduce cellular stress, playing a key role in the prevention of NAFLD exacerbation. Moreover, the histological characteristics of NASH may be improved by vitamin E, whilst immunomodulation, anti-inflammatory and anti-fibrotic properties are typical of vitamin Ds [112].

MD is also characterized by carotenoids intake. These comprise a class of natural fat-soluble pigments present in different types of fruits and vegetables, which act as antioxidants. Among them, lycopene, due to its potent antioxidant effect, has been reported as a potential protective agent in NAFLD.

As for the lipid lowering effects, intake of MUFA has been shown to prevent the development of NAFLD through an amelioration of plasma lipid levels and a decrease in body fat accumulation, as well as postprandial adiponectin expression. PUFAs are also responsible for the regulation of three major transcription factors involved in several pathways of liver metabolism of carbohydrates and lipids [113]. Beyond the improvement in steatosis, PUFAs can induce an independent anti-inflammatory effect by suppressing TNF-α and IL-6, responsible for the inflammation which occurs in NASH [114]. MD can also help in plasma cholesterol reduction through the high intake of water-soluble fibers commonly present in large concentrations in foods such as beans, vegetables, fruit and whole grains. Finally, water-soluble fibers are also able to the rate of biliary excretion by reducing both total and LDL cholesterol [115].

Randomized clinical trials on patients with NAFLD have shown a positive influence on glycemia [103,116]. In a recent Italian study on over 580 patients with various CV risk factors, adherence to the Mediterranean diet was demonstrated to allow both a reduced incidence of NAFLD and lower IR [117]. Moreover, the trial by Estruch et al. on about 7500 patients with high CV risk has demonstrated a lower incidence of cardiovascular events in patients assigned to the Mediterranean diet supplemented with extra-virgin olive oil as compared to a control population with a low-fat diet [118].

The Mediterranean diet is also able to affect the MS, as demonstrated by Kesse-Guyot et al., who demonstrated MD benefits on some components of MS such as waist circumference, systolic blood pressure and triglycerides [119].

### 7.3. Physical Activity

Physical activity is among the cornerstones of NAFLD management, even though the evidence behind its real usefulness is somewhat less solid than other conditions such as T2DM and CV risk [120,121].

Physical exercise, in terms of either aerobic, resistance or high intensity intermittent workout, seem to exert comparable outcomes on fatty liver. More dynamic aerobic exercise does not look like to provide an additional advantage over aerobic exercise, while continuity in physical activity is crucial [122,123].

The mechanisms underlying the change in fatty liver after exercise reflect changes in energy balance, circulating lipids, and insulin sensitivity. Physical exercise slightly affects hepatic insulin sensitivity, though improving peripheral insulin sensitivity and, consequently, reduction of de novo hepatic lipogenesis. However, recent studies show a direct effect of exercise on the increase in VLDL, which contributes to the reduction of liver fat. Moreover, the reduction of visceral fat also positively affects both inflammation and fibrosis, regardless of IR and hepatic steatosis [103,124].

Finally, it was demonstrated how much physical exercise must be associated with weight loss, responsible for up to 80% reduction in liver fat [125]. In fact, in a recent cross-sectional study by Bullón-Vela et al. on over 300 participants, the synergy between the MD, consumption of legumes and physical activity has been demonstrated to reduce non-invasive indicators of NAFLD [126].

## 8. Genetics and Epigenetics

According to the “multiple parallel hits hypothesis” theory, NAFLD has a multifactorial pathogenesis and, among the various factors, genetics plays an essential role. Single nucleotide polymorphisms (SNPs) of patatin like phospholipase domain-containing protein 3 (PNPLA3) have been largely demonstrated to exert a crucial role in both the development and progression of NAFLD. PNPLA3 gene is located on the long arm of chromosome 22 and its variant I148M (rs738409 C/G) is related to NAFLD development [127]. The variant I148M consists of a guanine in place of a cytosine, which transforms codon 148 from isoleucine to methionine [128].

PNPLA3 gene is responsible for the production of an intracellular lipase named adiponutrin, in turn involved in the hydrolysis of triglycerides inside the adipocytes [129] and its activity is reduced by up to 80% in subjects with variant I148M [130]. This favors the accumulation of intracellular lipids in the liver and reduction of very low-density lipoproteins (VLDL) [131]. PNPLA3 is mainly located in the ER and on the surface of lipid droplets in hepatocytes, adipocytes and HSC [132,133]. The mechanism by which the I148M variant induces the development of steatosis seems related to the accumulation of the mutated PNPLA3 protein. This might occur because of the lower accessibility to ubiquitin ligase and to the reduced proteasomal degradation of I148M protein components [134], which would hinder the activity of other lipases (e.g., PNPLA2), with a reduction in both turnover and mobilization of triglycerides [135].

In HSC the variant PNPLA3 I148M alters retinol secretion, thus contributing to fibrogenesis and carcinogenesis, with a consequent increase of the risk of cirrhosis and HCC development, regardless of the predisposition to steatosis [136,137,138]. The activation of the (SREBP1c)/(LXR) pathway induced by hyperinsulinemia and carbohydrate feeding transcriptionally modulate PNPLA3 expression.

A variant of TM6SF2 gene has been further reported to exert a role in the pathogenesis of NAFLD. The gene is located on the short arm of chromosome 19 and produces a homonymous protein of 351 amino acids [139]. TM6SF2 is mainly found in the ER and in the intermediate compartment of the Golgi reticulum [140], and it is mainly expressed in liver, kidneys and small intestine. The polymorphism C > T rs5854026 determines the replacement of glutamate with a lysine in residue 167 (E167K), with a reduction of protein function up to 46% [141]. In experimental models, this SNP produces a reduction in the secretion of VLDL, which causes the accumulation of triglycerides in the lipid droplets and, consequently, NAFLD [140,141]. Other functions of TM6SF2 are the synthesis of cholesterol [142], the mobilization of lipids for the assembly of VLDL [143] and the reduction of serum alkaline phosphatase activity [141]. People with E167K polymorphism in the liver tend to progress more often to NASH and to higher degrees of both steatosis and fibrosis.

Also, MBOAT-7 gene is involved in NAFLD pathogenesis. MBOAT-7 encodes a member of the O-acyltransferase family, integral membrane proteins with acyltransferase activity. The encoded protein is a phosphatidylinositol acyltransferase involved in the re-acylation of phospholipids through acyl-CoA transfer to phosphatidylinositol and other phospholipids, using arachidonoyl-CoA as preferred substrate [144,145]. The mutation of MBOAT-7 gene results in an increase of diacylglycerol, a precursor of triglycerides, in addition to collagen deposition, thus producing an accumulation of lipids and progression to fibrosis [146]. The reduced expression of MBOAT-7 is also related to NAFLD development due to the suppression of gene expression by the hyper-insulinemic states. This causes accumulation of phosphatidylinositol and an increase in the absorption of long-chain fatty acids in insulin-sensitive tissues [147]. Subsequently saturated triglycerides accumulate in hepatocytes due to reduced incorporation of arachidonic acid into phosphatidylinositol, consistent with the reduced enzymatic activity [144,145,148].

A further gene involved in the development of NAFLD is that encoding for the glucokinase regulatory protein (GCKR), which is involved in the regulation of the influx of glucose in the hepatocytes and de novo lipogenesis [149].

Beyond the various genetic components, also epigenetics plays a role in NAFLD development. Gene expression follows an epigenetic modulation typically characterized by: (a) DNA methylation; (b) remodeling of chromatin (histone modifications), and (c) construction of microRNA (small non-coding RNA molecules with specific functions).

Some nutrients (e.g., choline, methionine, folic acid and vitamin B12) are considered “methyl donors”, as they promote DNA and histone methylation. Methylation consists in adding a methyl group to a cytosine directly followed by a guanine (i.e., CpG sites). The methylation state of these sites may largely affect gene activity/expression due to the association between hypermethylation and gene repression and of hypomethylation with gene activation. Methylation is considered crucial in the progression from simple steatosis to NASH as well as in triglycerides metabolism, and it can be impaired by nutritional deficiencies of choline, betaine, vitamin B12, and folic acid. In fact, there is an association between the integration of these compounds and an increased hepatic efflux of triglycerides [150]. Conversely, if the organism is deficient of these substances, hepatic triglycerides may accumulate, thus favoring the overexpression of the genes involved in fatty acids synthesis [151].

Moreover, in humans with overt NAFLD, PPARs involved in the regulation of the expression of genes implicated in lipid and glucose metabolism were reported as hypermethylated, while genes encoding TGF-1 and PDGF hypomethylated [152]. In addition, in animal models, if methyl-donor micronutrients are introduced into a diet rich in fat and sucrose, this will allay the effect on the accumulation of triglycerides in the liver through the hypermethylation of a key enzyme for the synthesis of fatty acids [153]. Also, in vivo studies have shown the epigenetic effect of these nutrients, whose deficiency leads to an alteration of the function of endothelial cells and inflammatory processes, with consequent formation of plaque and an increased cardiovascular risk [154,155].

Another factor crucial for the definition chromatin architecture, hence gene accessibility, is the post-translational modification of histones. Main modifications are represented by acetylations, which are linked to gene transcription activation and catalyzed by histone acetyltransferases (HAT) and deacetylations, which are engaged indeed in gene repression and catalyzed by enzymes named histone deacetylases (HDAC). Particularly sirtuins (SIRT) form a group of proteins with deacetylase activity, with SIRT1 as key enzyme involved in DNA repair, histone and non-histone deacetylation induction of genes involved in IR and inflammation of adipose tissue. All these effects will promote gluconeogenesis and oxidation of fatty acids in the liver [156]. An association has been observed between NAFLD and either the reduced expression of SIRT1 or its reduced activity [157].

An inhibitor of histone acetyltransferase, tannic acid, a polyphenol of vegetal origin, has been reported to hinder histones’ hyperacetylation of the de novo lipogenesis promoter, thus reducing its activity and, consequently, the accumulation of hepatic fat [158]. SFAs (saturated fatty acids), on the other hand, inhibit histone deacetylase, with subsequent impairment of metabolic and inflammatory processes [159].

Finally, mRNA stability may be altered by the formation of small RNA molecules named microRNAs, which bind to transcriptional mRNA, and may consequently lead to post-transcriptional repression of targeted protein-coding genes. The most expressed in human liver is miR-122 [160]. Actually, miR-122 silencing occurs early during hepatocarcinogenesis starting from NASH and it could thus be considered as a new molecular marker of HCC risk assessment in patients with NASH [161]. miR-122 seems also responsive to diet in both in vitro and in vivo models and in humans. In particular, miR-122 appears upregulated in the presence of a high-fat diet, thus resulting in the promotion of lipogenesis in the liver [162].

## 9. Endothelial Dysfunction

NAFLD is a component of MS, as well as a risk factor for the development of CV diseases, regardless of diabetes, hypertension and obesity co-presence [163,164,165]. In fact, most of the deaths occurring in NAFLD patients might be due to CV causes [166]. The connection between NAFLD and cardiovascular disease may be observed in the alterations induced by metabolic liver diseases on the endothelial function, regardless of other CV risk factors.

The endothelium can also be thought as an organ crucially involved in vascular homeostasis, due to the release of a large number of substances with autocrine and paracrine activity (e.g., NO, prostacyclin, EDHF (hyperpolarizing factor of endothelial derivation), endothelin1, thromboxane A2, prostaglandin a2, PAF,…) [167]. These factors regulate the maintenance of vascular tone, vascular permeability, the balance between coagulation factors and fibrinolysis, as well as the composition of the subendothelial matrix and the process of proliferation/apoptosis of smooth muscle cells [168]. If the endothelium undergoes both a functional and physical damage, the homeostatic mechanisms fail, especially vasodilation linked to the release of NO, which consequently results in endothelial dysfunction (ED). ED is typically described by a reduced bioavailability of vasodilator molecules and/or an increase in vasoconstrictor stimuli such as thromboxane A2, prostaglandins H2, and ROS [169]. In patients with NAFLD several alterations of the carotid media-intimal thickness, atherosclerosis, calcification of coronaries and low reserve of coronary flow, as well as the degree of these vascular changes were reported as directly related to the harshness of the histological damage to the liver, outlined by lobular inflammation and extension of fibrosis [163,170,171]. In 2015 Long et al., on over 2200 patients without overt CV diseases, demonstrated an association between NAFLD (defined by the decrease in liver attenuation highlighted at CT) and abnormalities concerning microcirculation and endothelial dysfunction [172].

The correlation between NAFLD and microvascular dysfunction could derive because NAFLD, inducing the production of proinflammatory cytokines and creating low-grade inflammation, would lead to an inefficiency of the mechanisms underlying the endothelial homeostasis functioning [173]. The pathogenetic connection between NAFLD and ED can also be considered in the opposite way, as reported in several studies. ED would be able to induce and worsen metabolic liver disease, thus producing a vicious circle which feeds itself. ED may also be described as an early alteration in metabolic liver disease, which develops before any CV disease or any structural alteration of the vessel wall [174]. In a paper by Pasarìn et al., mice fed for 30 days with a diet consisting of 65% fat, especially saturated fats, were reported to develop early ED, prior to the development of structural endothelial modifications, inflammation and liver fibrosis [175]. An increase in portal perfusion pressure was observed in the liver of these mice, as compared to those fed with the conventional diet. This difference was reduced using NO, hence the most likely hypothesis is that this condition to the portal flow could be due to an increase in the vascular tone [175]. Moreover, in the mice of the experiment levels of phosphorylated nitric oxide synthetase (NOS), hence active, were lower, to then produce an increase in NOS active form levels after insulin administration, thus indicating that during the first phase of ED insulin resistance was involved in the mice fed with 65% saturated fat. Finally, a correct production of NO due to suitable activation of NOS was reported to halt the activation of hepatic stellate cells and prevent sinusoidal thrombosis, a known progression mechanism of liver cirrhosis [175,176].

Therefore, a potential link between NAFLD and CV disease was identified in ED. Due to this reason, the elaboration of new diagnostic approaches to measure ED would be worthied for a better prediction of CV risk in patients with NAFLD. In this regard, invasive methods (e.g., intravascular injection of acetylcholine, measurement of vasodilation caused by this neurotransmitter,…) and non-invasive methods, unsustainable for ED screening from a financial point of view (flow mediated dilation), may be used to assess for ED, till the serum assay of ED markers such as Endocan (ESM-1), a soluble proteoglycan produced by endothelial cells, discovered by Lassalle and collaborators in 1996 [177]. ESM-1 is released by damaged endothelial cells in response to proinflammatory stimuli and angiogenetic factors [178,179]. Moreover, HMGB1 molecule is strictly involved in the inflammatory response regulation of endothelial cells, as well as in the process of endothelial repair and homeostasis [180]. In patients with NAFLD and coronary artery disease (CAD), ESM-1 has been shown as higher than among controls, and its level is directly related to the severity of CAD, observed by arteriography. Indeed, lower HMGB1 levels were reported in patients with both NAFLD and CAD rather than in NAFLD subjects without CAD. As well, there was a significant reduction of HMGB1/ESM-1 ratio in NAFLD CAD patients as compared to controls. Such a finding might stand in the alteration of the balance between damage (measured by ESM-1 levels) and endothelial repair (assessed by HMGB1 levels). Therefore, ED identification in patients with simple steatosis, with low-cost methods, can provide additional information on patients’ prognosis, besides the evaluation of conventional risk factors, both for the CV system and the liver. In this sense, the scientific community has been looking for new therapeutic strategies for NAFLD to act at an earlier stage of the disease and halt ED.

## 10. Clinical Impact

NAFLD and MAFLD are acronyms as for hepatic involvement in the MS. Thus, their clinical impact is represented by hepatic and extra-hepatic involvement, and the related complications may further increase mortality as compared to the healthy population [181]. NAFLD is currently considered the main chronic non-viral liver disease, even more fearful than HCV and HBV infections, for which the effectiveness of antiviral therapies and vaccines have significantly improved the prognosis [182,183,184,185,186,187].

The advent of chronic inflammation (NASH) in the steatosic liver represents the first step towards a potential progression into fibrosis and, subsequently, cirrhosis. Such an event is silent and inevitably leads to complications, first of all HCC, often observed even in the stages of pre-cirrhotic fibrosis [188,189,190]. The impact of NAFLD-related liver disease has also been triggering an increasing proportion of patients undergone to liver transplantation and consequent immunosuppressive therapies [191,192]. In the absence of effective pharmacological therapies, screening and clinical-instrumental monitoring currently represent the sole instruments available to constrain. Loomba and colleagues retrospectively observed on over 10 million of individuals a 5.7% prevalence of NAFLD, of which 71.1% NAFLD/NASH and 28.9% NAFLD-cirrhosis. Risk factors for progression have been identified in CV disease, renal impairment, dyslipidemia and diabetes [193]. Hence, liver ultrasound (US) and the quantification of fat and fibrosis with transient elastography (Fibroscan) and Control Attenuation Parameter (CAP) software currently represent the non-invasive and inexpensive tools to diagnose NAFLD in the clinical practice [194]. Beyond these, also the evaluation of genetic polymorphisms (PNPLA3, TM6SF3, MBOTT) and scores (e.g., NAFDL fibrosis score, FIB4, APRI score) may support the identification of subjects at higher risk of disease progression [195]. The histological evaluation of liver tissue by US-guided biopsy is an invasive method which allows for an effective assessment of the degree of progression of fibrosis and inflammation. Hence, it should be taken into consideration when the clinical-instrumental and laboratory indices suggest an evolution towards the progressive phase.

NAFLD and IR are bidirectionally correlated and, consequently, the development of pre-diabetes and diabetes is the most direct consequence at the extrahepatic level. In turn, T2DM is a well-known risk factor for multiorgan damage, including an involvement, among others, of CV system, kidney and peripheral nervous system [155,196,197,198,199,200,201,202]. Obesity and MS are the two side of the same coin, as steatosis represents their liver involvement [203]. Therefore, several clinical studies have found an association of severe steatosis and coronary heart disease with an increased incidence of CV mortality [204,205,206,207,208]. The higher CV risk is also associated with an increased mortality rate, as confirmed by two recent studies [209,210]. The severity of fibrosis related to steatosis plays an important role in the assessment of CV risk. Non-invasive investigations allow to obtain a reliable and predictive evaluation [211,212].

Likewise, the association of NAFLD and subclinical atherosclerosis characterized by the involvement of carotid branches, a prelude to cerebrovascular ischemic events, has been clearly assessed. Moreover, in these cases the noninvasive examinations by Doppler US are easy to use and inexpensive tools in the clinical practice. Doppler US allows the identification of major vascular wall alterations such as impaired endothelium-dependent flow-mediated vasodilatation, carotid artery intima-media thickness (IMT) and an increased arterial wall stiffness [213,214,215].

Similarly to the association between NAFLD and coronary heart disease, several studies have shown a clear increase in the risk of cerebrovascular events (stroke) and NAFLD as compared to the general population. Such events more closely related to high fibrosis grade show both a greater severity and prognosis than cerebrovascular events occurring in the non-NAFLD population [216,217,218].

Renal function plays a fundamental role in body detoxification, secretion of some hormones and water-electrolyte balance [219,220,221]. Therefore, the evaluation of its functional integrity always exerts a primary function in all chronic diseases. The possible interaction between chronic kidney disease (CKD) and NAFLD has been largely studied, even though it is not always easy to define a clear association between these two conditions. Some factors shared by both diseases such as T2DM can render their interpretation much more difficult [222,223,224,225]. A case-control study on over 48,000 patients both with and without NAFLD (ratio 1:1) has shown a significantly higher prevalence of CKD in NAFLD group (17.1 versus 11.6%) [226]. A cross-sectional study instead diversified the prevalence of CKD (classified as either stage 1–3 or abnormal albuminuria) in the diagnosis of NAFLD and MAFLD in over 12,000 patients. The authors showed a prevalence of NAFLD and MAFLD of 36% and 30%, respectively, with a greater reduction in glomerular filtrate and a greater prevalence of CKD in MAFLD [227]. A recent meta-analysis by Mantovani et al. examined over 122,000 patients with a NAFLD prevalence of 28% and almost 34,000 cases of incident CKD with stage 3 or higher monitored for a mean follow-up of 9 years. The authors found a risk of CKD in NAFLD 1.43 times higher, regardless of risk factors such as age, sex, obesity, hypertension, diabetes [228].

## 11. Conclusions

The increase in the incidence of MS in both industrialized and developing Countries, above all due to changes in diet and lifestyle, is associated with an equally significant increase in NAFLD. This trend leads to an increase in both morbidity and mortality due to both metabolic, hepatic and CV diseases.

Therefore, the slowdown in the increase of the “bad company” constituted by MS and NAFLD, with all the consequent direct and indirect costs, represents one of the main challenges for the National Health Systems worldwide.

## Abbreviations

NAFLD	Non-alcoholic fatty liver disease
MS	Metabolic syndrome
IR	Insulin resistance
CV	Cardiovascular
HDL	High density lipoproteins
FFAs	Free fatty acids
ER	Endoplasmic reticulum
PDFF	Proton density fat fraction
MRI	Magnetic resonance imaging
NASH	Non-alcoholic steatohepatitis
HCC	Hepatocellular carcinoma
T2DM	Type 2 diabetes mellitus
BMI	Body Mass Index
OGTT	Oral glucose tolerance test
HbA1c	Glycated hemoglobin
MAFLD	Metabolic associated fatty liver disease
IRS	Insulin receptor
PI3K	Phosphatidylinositol 3-kinase
GLUT-4	Glucose transporter protein type-4
TNF-α	Tumor necrosis factor-alpha
SREBP-1/2	Sterol regulatory element-binding protein-1/2
ChREBP	Carbohydrate-responsive element-binding protein
PPAR-γ	Peroxisome proliferator-activated receptor gamma
BA	Bile acids
LPS	lipopolysaccharides
SCFA	Short-chain fatty acids
ROS	Reactive oxygen species
O2^•−^	superoxidation radicals
H_2_O_2_	Hydrogen peroxide
NADPH	Nicotinamide adenine dinucleotide phosphate hydrogen
NF-κB	Nuclear factor kappa B
JNK	Jun *N*-terminal kinase
IKK	Inhibitor of nuclear factor kappa-B kinase
EASL	European Association for the Study of Liver
AASLD	American Association for the Study of Liver Disease
PUFA	Polyunsaturated fatty acid
MD	Mediterranean diet
MUFA	Mono-unsaturated fatty acid
IL	Interleukin
LDL	Low density lipoprotein
VLDL	Very low density lipoprotein
SNPs	Single nucleotide polymorphisms
PNPLA3	Patatin like phospholipase domain-containing protein 3
HSC	Hepatic stellate cells
LXR	Liver X receptor
MBOAT-7	Membrane Bound O-Acyltransferase Domain Containing 7
GCKR	glucokinase regulatory protein
TGF-1	Transforming growth factor beta 1
PDGF	Platelet-derived growth factor
HAT	Histone acetyltransferase
HDAC	Histone deacetylases
SIRT	Sirtuins
SFAs	Saturated fatty acids
EDHF	hyperpolarizing factor of endothelial derivation
ED	endothelial dysfunction
NOS	Nitric oxide synthetase
ESM-1	Endocan
HMGB1	High mobility group box 1 protein
CAD	coronary artery disease
HCV	hepatitis C virus
HBV	hepatitis B virus
US	ultrasound
CAP	Control attenuation parameter
IMT	Intima-media thickness
CKD	Chronic kidney disease
PIP3	Phosphatidylinositol (3,4,5)-trisphosphate
aPKC	activated protein kinase C
AKT1	Protein kinase B
GSK3	Glycogen synthase kinase 3
mTOR	Mammalian target of rapamycin
FOXO1	Forkhead box protein O1
PDK1	3-Phosphoinositide-dependent protein kinase 1
P	phosphorylated
